# Economic impact of health insurance fraud, waste, and abuse: a literature review of direct and indirect costs and system sustainability

**DOI:** 10.3389/fpubh.2026.1883020

**Published:** 2026-07-30

**Authors:** Naof F. Saleem Al-Ansary

**Affiliations:** Department of Public Health, College of Public Health, Imam Abdulrahman Bin Faisal University, Dammam, Saudi Arabia

**Keywords:** abuse, control interventions, economic impact, fraud, health insurance, sustainability, waste

## Abstract

**Purpose:**

This study examines the economic impact of fraud, waste, and abuse (FWA) in health insurance systems and synthesizes evidence on governance, legislative, regulatory, and technology-based interventions aimed at improving financial sustainability. It further evaluates the implications of these interventions for healthcare system resilience and identifies research gaps to inform future policy and practice.

**Method:**

This study employed a systematic literature search followed by a narrative synthesis to examine the economic impact of health insurance FWA. A comprehensive search of Scopus, Web of Science, MEDLINE, Embase, PubMed, OpenAlex, and Dimensions was conducted for studies published up to 24 November 2025, complemented by backward and forward citation tracking using the Inciteful.xyz platform. Following screening and eligibility assessment, 30 studies were included in the final narrative synthesis.

**Results:**

The findings indicate that FWA imposes substantial direct and indirect economic costs on health insurance systems by increasing financial losses, reducing operational efficiency, weakening accountability, and threatening long-term fund sustainability. Evidence suggests that effective mitigation requires an integrated approach combining preventive monitoring, robust governance, legislative enforcement, whistleblower protections, and advanced analytical technologies. Artificial intelligence, machine learning, blockchain, and automated auditing systems demonstrated promising performance in improving fraud detection under experimental conditions; however, evidence supporting their effectiveness in large-scale operational healthcare settings remains limited, and implementation challenges related to scalability, interoperability, privacy, and governance should be considered. Publication trends indicate increasing international research interest in FWA, although evidence remains concentrated in a limited number of countries.

## 1 Introduction

### 1.1 The universality of the healthcare FWA issue

Universal Health Coverage plays a pivotal role in healthcare delivery by aiming to provide societies with equitable access to healthcare and protection against catastrophic financial hardship. This movement gained traction from its inception under the Alma-Ata Declaration in 1978, up until the relatively recent launching of Sustainable Development Goal 3. Given this contemporary landscape, it is safe to say that healthcare systems continuously face the challenge of leveraging capabilities to meet the ever-growing demand.

Health insurance schemes are among the most commonly used financial protection strategies for covering healthcare costs. The World Health Organization (2000) asserts that health insurance plays a crucial role as an essential component of every health system (1). It assumes a substantial portion of system responsibilities and profoundly affects health outcomes. Nonetheless, as with other insurance systems, health insurance is susceptible to exploitation and fraud. FWA practices are costing patients and taxpayers billions of dollars, at a time when resources are already falling short of meeting the rapidly growing demand. It is estimated that 3 to 10% of total healthcare spending is lost due to fraud and abuse, resulting in billions of dollars in losses each year (2). Worldwide, health insurance systems incur substantial financial losses due to fraud and misuse. Health insurance fraud is a substantial global issue, with average global losses estimated at $455 billion in 2015, or 6.19% of total health expenditures (3). In the United States, for example, the National Health Care Anti-Fraud Association estimates annual losses of $100 to $170 billion, accounting for approximately 3 to 15% of the total healthcare budget (4). Another study estimates that waste-related costs in the US healthcare system range from $760 billion to $935 billion, accounting for approximately 25% of total healthcare spending from 2012 to 2019 (5).

A primary challenge in health insurance management is preventing fraud and abuse (6, 7). The complexity of healthcare delivery, heterogeneity of data, and the involvement of multiple stakeholders (patients, providers, and insurers) make effective monitoring challenging. Fraud, waste, and abuse, as examined in this review, encompass a variety of perpetrators and forms, e.g., patient, provider, and prescription fraud.

### 1.2 The importance of healthcare funds’ sustainability

Sustainability has been a concern for both governments and the healthcare sector, as it affects the health system, patient well-being, and the community at large. Here, the economic sustainability of healthcare means that systems, such as health insurance, can remain financially viable over time while delivering good care without reducing coverage. This is significant since it ensures that individuals do not have to fret about expenditures, as they can access the medical services they require. In health insurance, the degree to which funds are raised, pooled, and utilized will determine sustainability. This balance is threatened by FWA practices which divert resources from actual healthcare needs. Such practices reduce the efficiency of insurance systems as a whole by increasing costs, inflating claims, and reducing overall efficiency. They also harm insurers, consumers, and the economy at large by increasing costs, eroding trust, and wasting resources.

## 2 Definitions of fraud, waste, and abuse

The health insurance system comprises three key groups: the insured, medical institutions, and insurance companies. The differing interests of these groups may lead to FWA. Villegas-Ortega et al. (8) characterize health insurance fraud as an act of deceit or deliberate misrepresentation to obtain illicit advantages in insurance coverage. Waste, on the other hand, is defined as the utilization or misuse of resources in a manner that is more expensive but does not benefit patients. It is further described as actions that make processes less efficient and less effective. Examples include ordering unnecessary tests, prescribing brand-name medications rather than generics, and ordering costly procedures for routine care.

Healthcare abuse involves individuals acting in ways that cost more money, typically without an intention to deceive anyone. This includes charging too much or for services that were not actually provided. Such activities do not conform to standard financial practices and may result in inefficiencies and, in some cases, even constitute fraud. Lee and Cho (9) define abuse as medical care that does not adhere to sound fiscal practices, such as overtreatment or high charges.

### 2.1 Measures to control health care FWA

The diversity of interventions available in the literature to control FWA in healthcare ranges from the use of AI to the introduction of government-restrictive statutes to the empowerment of whistleblowing cultures. Approaches to detecting FWA differ markedly across countries. In the United States, the emphasis has been on combining statistical and regulatory tools such as multivariate outlier detection, quantile-based selection models, and accounting-driven regulation alongside enforcement initiatives and a growing reliance on data mining, analytics, machine learning, blockchain, and multi-stage algorithmic systems (10–18). The literature published on China reflects a stronger orientation toward computational techniques, including multi-view bi-clustering, extraction algorithms, and broader data mining approaches, complemented by risk governance frameworks and policy measures (19–23). In Saudi Arabia, the shift has been toward integrating newer digital infrastructures like blockchain, 5G, cloud systems, and machine learning into FWA detection (24, 25), whereas the UAE has concentrated more specifically on blockchain and its integration with large language models (25, 26).

In other countries, the approaches are more varied, but they still provide useful insights. Countries like Ireland have explored ontology-guided policy information extraction (27), Ghana’s work combines data mining with machine learning (28), while Pakistan has moved toward AI-based frameworks (5). In South Asia, India and Bangladesh have both turned to blockchain (29, 30).

### 2.2 The impact of FWA on healthcare sustainability

Efforts to control FWA in health insurance shape how efficiently systems use money, how fair they are, and whether they can last over time. When detection and prevention improve, financial losses decrease, and resource use becomes more balanced and reliable.

In the United States, these efforts have reduced waste, recovered substantial sums through fraud detection, removed biased incentives, supported the movement toward universal health coverage, and lowered annual financial losses (10, 12, 31). In China, the results are seen in better management of medical insurance funds, with improved efficiency, stronger equity, and more stable long-term sustainability (19, 22). In Saudi Arabia, these measures have helped lower insurance premiums and reduce annual financial losses. In other countries, the approaches differ, but the effects are still clear. Iran has improved the accuracy and efficiency of prescription fraud detection. Bangladesh has made claim processing faster, more secure, and more reliable. The UAE has reduced annual financial losses, while Indonesia continues to deal with financial inefficiencies that affect the sustainability of its national health insurance program. Pakistan has improved the allocation of healthcare resources, and the United Kingdom has strengthened the economic sustainability of its healthcare system (Haddad Soleymani et al. [32); (5, 25, 29, 33, 34)]. Unlike previous reviews that have primarily examined individual fraud detection methods or specific policy interventions, this review adopts an integrated perspective by synthesizing evidence on the economic burden of fraud, waste, and abuse (FWA) together with legislative, governance, institutional, and technology-driven control strategies. By combining evidence from diverse healthcare systems, the review evaluates how legal enforcement, institutional governance, artificial intelligence, machine learning, blockchain, and analytical approaches collectively influence financial sustainability and health system resilience. This multidisciplinary perspective provides policymakers, healthcare administrators, insurers, and researchers with a comprehensive understanding of how effective FWA control contributes to the long-term sustainability of health insurance systems and supports progress toward universal health coverage.

## 3 Methodology

### 3.1 Materials and methods

This review employed a systematic literature search followed by a narrative synthesis to examine the economic impact of fraud, waste, and abuse (FWA) in health insurance systems. A systematic approach was adopted to identify, screen, and select relevant studies, and the review process was reported in accordance with the PRISMA 2020 guidelines to enhance transparency and reproducibility. The included studies exhibited substantial heterogeneity in terms of research designs, healthcare settings, intervention types, and outcome measures; therefore, a quantitative meta-analysis was not considered appropriate. Instead, the evidence was synthesized narratively by identifying recurring themes and organizing the findings into major domains, including the economic burden of FWA, governance and regulatory interventions, technological and analytical approaches, and implications for healthcare sustainability.

A comprehensive search strategy was developed in consultation with a subject expert and implemented across seven electronic databases: Scopus, Web of Science, MEDLINE, Embase, PubMed, OpenAlex, and Dimensions. These databases were selected to ensure broad coverage of multidisciplinary literature related to health insurance fraud, healthcare financing, governance, and emerging fraud detection technologies. The literature search included all eligible studies published up to 24 November 2025. To maximise retrieval of relevant evidence, backward and forward citation tracking was conducted using the Inciteful.xyz platform.

Following retrieval, duplicate records were removed prior to screening. Titles and abstracts were then screened against predefined eligibility criteria to identify potentially relevant studies. Articles considered eligible underwent full-text assessment to determine their relevance to the objectives of the review. Studies were included if they examined the economic impact of FWA in health insurance systems, evaluated fraud detection or prevention interventions, or reported implications for healthcare financing, governance, or system sustainability. Studies published as conference abstracts, editorials, commentaries, case reports, or in languages other than English were excluded. After completion of the screening and eligibility assessment, 30 studies met the inclusion criteria and were included in the final narrative synthesis.

### 3.2 Search strategy

A comprehensive and systematic search strategy was developed in consultation with a subject expert to identify studies examining the economic impact of fraud, waste, and abuse (FWA) in health insurance systems. The search strategy was designed around three core concepts: (i) health insurance and healthcare financing, (ii) fraud, waste, and abuse, and (iii) economic impact and sustainability. Controlled vocabulary (where available) and free-text keywords were combined using Boolean operators (AND, OR) to maximize both the sensitivity and specificity of the search.

Because indexing systems and search functionalities differ across bibliographic databases, the search syntax was adapted to the requirements of each database while maintaining the same conceptual framework. Specifically, TITLE-ABS-KEY fields were used in Scopus, Topic (TS) searches were used in Web of Science, and Medical Subject Headings (MeSH) were combined with keyword searches in PubMed and MEDLINE. Equivalent search fields, indexing terms, truncation symbols, and controlled vocabularies were used in Embase, OpenAlex, and Dimensions to ensure comprehensive and consistent retrieval of relevant studies.

The search strategy consisted of the following three concept groups:

Concept 1: Health insurance and healthcare financing.

(“health economic*” OR “healthcare economic*” OR “health care economic*” OR “healthcare finance*” OR “health care finance*” OR “health financing” OR “healthcare financing” OR “health care financing” OR “healthcare funding” OR “health care funding” OR “healthcare sustainab*” OR “health system sustainab*” OR “financial sustainab*” OR solvency OR “budget impact” OR expenditure* OR spending OR “economic burden”)

AND

Concept 2: Fraud, waste, and abuse.

(“health insurance fraud” OR “medical insurance fraud” OR “insurance fraud” OR “improper payment*” OR overpayment* OR “false claim*” OR “fraudulent claim*” OR upcod* OR overbilling OR miscoding OR “phantom billing” OR “ghost billing” OR “provider fraud” OR “consumer fraud” OR “organized fraud” OR “organised fraud” OR “organized fraudulent behaviour” OR “organised fraudulent behaviour” OR “duplicate charge*” OR “duplicate claim*” OR overestimation OR underestimation)

AND

Concept 3: Economic impact and sustainability.

(“direct cost*” OR “indirect cost*” OR “financial loss*” OR “fraud loss*” OR “economic burden” OR “budget impact” OR sustainab*)

The complete database-specific search strategies, including database-specific syntax and field tags, are provided in Supplementary Appendix A to facilitate transparency and reproducibility.

To ensure comprehensive coverage of the literature, backward and forward citation tracking was performed using the Inciteful.xyz platform. In addition, the reference lists of all eligible studies were manually screened to identify potentially relevant publications that were not retrieved through the electronic database searches.

**Figure 1:**
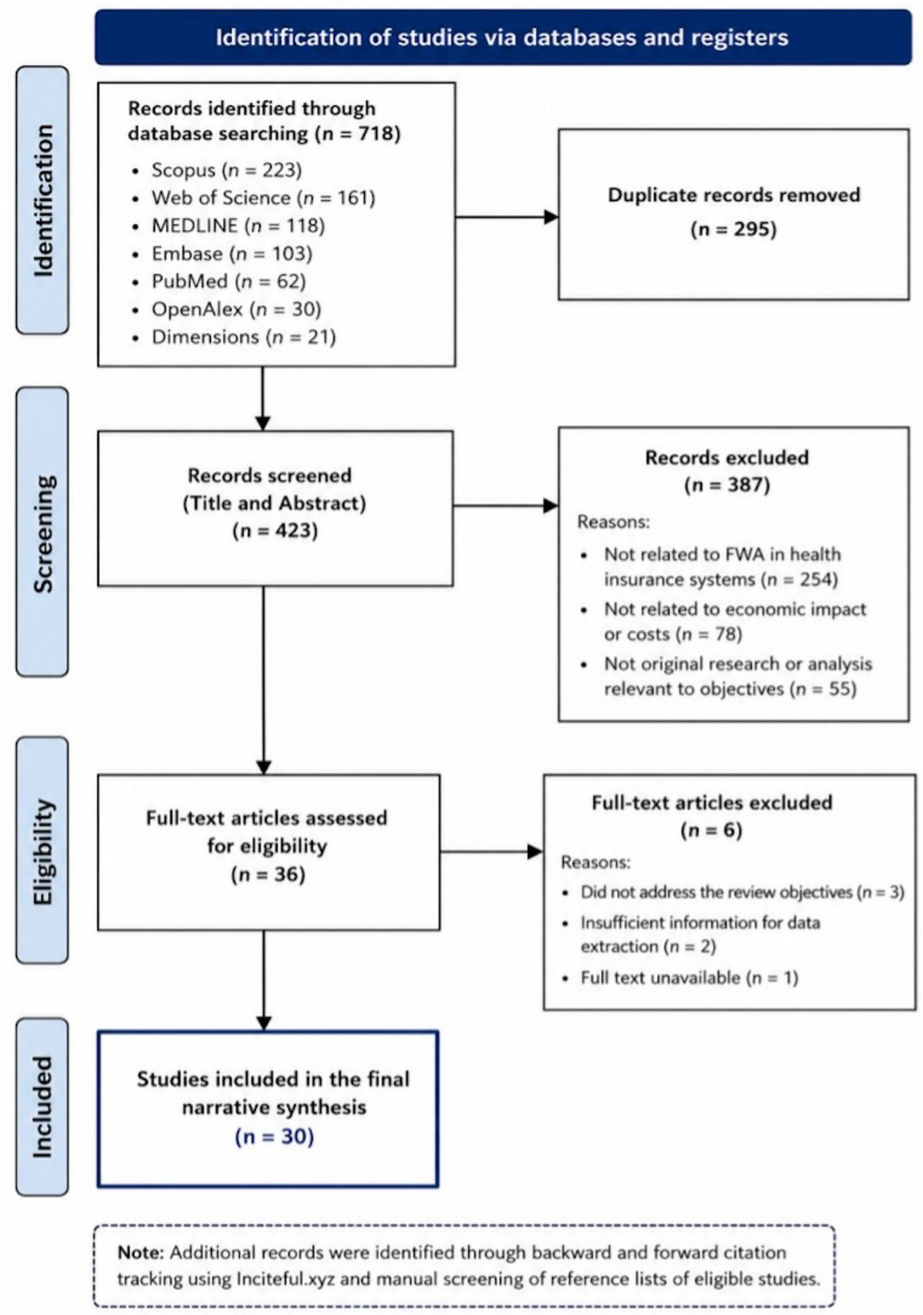
PRISMA 2020 flow diagram illustrating the systematic literature identification, screening, eligibility assessment, and study selection process. Diagram adapted from page et al. (48) under the terms of the creative commons attribution license (CC BY 4.0).

The systematic search identified 718 records across the seven electronic databases. After removing 295 duplicate records, 423 unique records remained for title and abstract screening. Records that did not satisfy the predefined eligibility criteria were excluded at this stage. The remaining potentially eligible studies underwent full-text assessment for eligibility. Following the full-text review, 30 studies met all inclusion criteria and were included in the final narrative synthesis. The complete study selection process is presented in the PRISMA 2020 flow diagram (Figure 1).

### 3.3 Study selection and eligibility

Following the removal of duplicate records, titles and abstracts were screened against the predefined eligibility criteria to identify potentially relevant studies. Articles considered potentially eligible subsequently underwent full-text assessment to determine their suitability for inclusion in the review. Studies were included if they examined the economic impact of fraud, waste, or abuse in health insurance systems, evaluated fraud detection or prevention interventions, or discussed implications for healthcare financing and system sustainability. Conference abstracts, editorials, commentaries, case reports, and studies published in languages other than English were excluded.

The screening and study selection process was undertaken by the primary reviewer in consultation with a subject expert. Any uncertainties regarding study eligibility were resolved through discussion until consensus was reached. Because this review employed a narrative synthesis of heterogeneous evidence rather than a quantitative meta-analysis, no numerical scoring or cut-off thresholds were applied during study selection. Instead, inclusion decisions were based on the predefined eligibility criteria and the relevance of each study to the objectives of the review.

### 3.4 Quality assessment

A formal methodological quality appraisal or quantitative risk-of-bias assessment was not undertaken because the review aimed to provide a broad narrative synthesis of heterogeneous evidence encompassing empirical studies, policy analyses, technological evaluations, and review articles. Given the diversity of study designs and outcome measures, the review focused on critically synthesizing the available evidence rather than quantitatively evaluating methodological quality. This limitation has been acknowledged in the Limitations section and should be considered when interpreting the findings.

### 3.5 Eligibility criteria

Studies were eligible for inclusion if they: (i) investigated fraud, waste, or abuse in health insurance or healthcare financing; (ii) examined economic costs, financial burden, sustainability implications, or fraud control interventions; (iii) were published in peer-reviewed journals in English; and (iv) provided sufficient methodological information to support interpretation of the findings. Studies were excluded if they consisted solely of conference abstracts, editorials, commentaries, or case reports, focused exclusively on clinical outcomes without addressing fraud-related economic issues, or lacked sufficient information for data extraction.

Because of the methodological and contextual heterogeneity across the included studies, the evidence was synthesized narratively by grouping studies according to economic impact, governance interventions, technological approaches, legislative measures, and healthcare sustainability outcomes.

## 4 Scope and objectives

This review synthesizes evidence on the economic impact of fraud, waste, and abuse (FWA) in health insurance systems and examines governance, legislative, regulatory, and technology-based interventions designed to mitigate their effects. Specifically, the review aims to: (i) evaluate the direct and indirect economic burden of FWA; (ii) identify and compare governance, legal, institutional, and technological approaches used to prevent and detect FWA; (iii) assess the implications of these interventions for the financial sustainability of health insurance systems; and (iv) identify research gaps and priorities for future investigation. The review encompasses a broad spectrum of fraudulent practices, including duplicate claims, upcoding, undercoding, overbilling, phantom billing, false claims, and prescription fraud.

## 5 Related works

Previous research consistently demonstrates that fraud, waste, and abuse (FWA) impose substantial financial burdens on health insurance systems worldwide, although estimates vary according to differences in healthcare systems, fraud definitions, and methodological approaches. Global estimates suggest that healthcare fraud accounts for a considerable proportion of healthcare expenditure, with reported losses ranging from approximately 3 to 15% of total healthcare spending depending on the country and estimation methodology (3, 35–37). In the United States alone, annual healthcare fraud losses have been estimated to range between US$10 billion and US$200 billion, while improper payments in federal health insurance programmes exceeded US$100 billion in 2023 (36). Similarly, Gee and Button (3) estimated global healthcare fraud losses at approximately US$415 billion, whereas Gee et al. (35) reported an average Percentage Loss Rate (PLR) of 5.59%, illustrating the substantial economic burden of FWA across different healthcare systems.

The literature further demonstrates that technological approaches have become increasingly important in fraud detection. Earlier studies primarily relied on statistical methods, data mining, clustering algorithms, decision trees, neural networks, and regression models to identify suspicious claims (38, 39). More recent investigations have expanded these approaches through artificial intelligence, machine learning, blockchain technologies, and automated analytical systems, reflecting a shift toward more sophisticated and data-driven fraud detection frameworks (25, 30, 40). Nevertheless, although these technologies have reported promising performance in experimental environments, evidence regarding their effectiveness in routine operational healthcare settings remains comparatively limited.

Beyond technological innovation, several studies emphasize that sustainable FWA control depends equally on governance, legislative enforcement, and institutional oversight. Regulatory reforms, stronger auditing systems, payment integrity programmes, whistleblower protections, and improved accountability mechanisms have all been associated with enhanced fraud prevention and greater financial sustainability (3, 36, 40). At the same time, micro-health insurance and stronger financial protection mechanisms have been shown to reduce catastrophic healthcare expenditure and improve access to healthcare among vulnerable populations, further highlighting the broader implications of effective health insurance governance (41).

Despite the growing body of literature, several important gaps remain. Most existing studies have focused on high-income countries, particularly the United States and China, whereas comparatively limited evidence is available from low- and middle-income countries. Furthermore, many recent studies evaluate AI- and blockchain-based fraud detection using retrospective datasets or proof-of-concept implementations, with relatively little evidence concerning large-scale implementation, long-term sustainability, or real-world effectiveness. These gaps provide the rationale for the present review, which synthesizes multidisciplinary evidence on the economic burden of FWA and critically evaluates governance, legislative, institutional, and technological interventions that support the sustainability of health insurance systems.

## 6 Findings

### 6.1 The economic burden of FWA on health systems

The evidence synthesized across the included studies demonstrates that the economic consequences of fraud, waste, and abuse extend well beyond direct financial losses. The reviewed literature consistently indicates that effective FWA control requires coordinated legislative, institutional, governance, and technological interventions rather than reliance on isolated fraud detection methods. Collectively, these approaches improve financial accountability, strengthen insurance fund sustainability, enhance operational efficiency, and contribute to broader health system resilience. The economic burden of FWA in health insurance is substantial and affects healthcare systems worldwide through direct financial losses and reduced availability of resources for essential public services. Globally, healthcare fraud and abuse are estimated to account for 6.19 to 7.3% of total health expenditure, amounting to nearly $740 billion by 2022. For example, in the United States, FWA added at least $98 billion to Medicare and Medicaid spending in 2011, while by 2014, around $60 billion was lost from Medicare funds due to incorrect billing, fraud, and abuse. Similar financial losses have been reported in developing countries. In Indonesia, nearly one million fictitious hospital claims worth Rp. 2 trillion were identified in 2016. Likewise, in Peru, annual losses faced by the main public insurer due to fraudulent activities are estimated to range from USD 18.6 million to USD 61.8 million (8, 11, 13, 33, 42, 43). As noted by Vian (43), “the costs of healthcare fraud extend beyond direct financial losses to include indirect effects such as weakened system efficiency, reduced accountability, and diversion of resources from essential health services.” This difference is significant because direct costs are quantifiable financial losses resulting from false claims and overpayments. In contrast, indirect costs are taken to represent administrative burden, increased compliance costs, and long-term inefficiencies within the health system (11, 42).

The issue of FWA is present universally; however, health systems react differently to address it. For example, some focus on improving detection tools by allocating budgets and establishing commission enforcement units. In contrast, others focus on passing laws and statutes that criminalize practices that lead to FWA. Furthermore, mature systems employ a multi-pronged strategy that addresses prevention, governance, and solvency to create a hospitable environment for financial sustainability.

Although estimates of the economic burden of fraud, waste, and abuse are consistently substantial, they should be interpreted cautiously because the included studies employed different definitions of FWA, healthcare financing systems, methodological approaches, and reporting frameworks. Consequently, reported financial losses are not directly comparable across countries and should be viewed as indicative estimates rather than precise global measures.

### 6.2 FWA control interventions

The reviewed literature demonstrates that no single intervention is sufficient to prevent fraud, waste, and abuse (FWA) in health insurance systems. Instead, the evidence consistently indicates that the most effective approaches combine robust governance, regulatory oversight, legislative enforcement, institutional accountability, and advanced analytical technologies. While governance and regulatory mechanisms establish the institutional environment necessary for fraud prevention, technological innovations improve the efficiency and accuracy of fraud detection. Collectively, these complementary approaches strengthen financial accountability, improve resource utilization, and enhance the long-term sustainability of health insurance systems.

#### 6.2.1 Governance, regulatory and legislative interventions

The literature consistently identifies governance, regulatory oversight, and legislative enforcement as the foundation of effective FWA control. Across the reviewed studies, institutional governance mechanisms including audits, prepayment reviews, payment integrity programs, and dedicated fraud investigation units were associated with improved fraud detection, reduced financial losses, and stronger insurance fund sustainability. Greater investment in Medicaid Fraud Control Units, together with strengthened institutional oversight, has been linked to improved fraud detection outcomes and healthier insurance fund balances, highlighting the importance of governance in maintaining long-term financial stability (12, 14, 22, 26, 44).

Evidence from the United States further demonstrates that legislative reforms can substantially strengthen fraud prevention when combined with institutional monitoring. The Affordable Care Act shifted fraud control from the traditional “pay-and-chase” approach toward preventive monitoring by expanding program integrity initiatives, increasing enforcement activities, and strengthening provider accountability. Similar governance reforms in China, including intelligent supervision systems and unannounced “flight inspections,” have improved policy implementation and fraud detection. Although these interventions have produced measurable financial benefits, their effectiveness depends on sustained regulatory capacity, institutional commitment, and continuous monitoring rather than legislation alone (23, 44).

#### 6.2.2 Technological and analytical interventions

The reviewed literature demonstrates that emerging digital technologies, including artificial intelligence (AI), machine learning (ML), blockchain, and advanced data analytics, have considerable potential to enhance fraud detection, claims verification, and decision support in health insurance systems. Across the included studies, these technologies consistently outperformed many conventional analytical approaches under experimental conditions, reflecting their ability to identify complex fraud patterns within large healthcare datasets. However, most of the available evidence is derived from retrospective datasets, simulated environments, or proof-of-concept implementations rather than routine operational healthcare settings. Consequently, the reported performance metrics should be interpreted cautiously, as they may not accurately reflect real-world effectiveness or scalability. Successful implementation depends not only on technological capability but also on interoperability with existing health information systems, organizational readiness, regulatory compliance, data quality, cybersecurity, patient privacy, ethical governance, and continuous human oversight. These findings suggest that technological innovation should complement, rather than replace, robust governance and institutional control mechanisms in achieving sustainable fraud prevention.

##### 6.2.2.1 Advanced machine learning and clustering methodologies

Machine learning has emerged as one of the most extensively investigated approaches for detecting health insurance fraud. Across the reviewed studies, supervised learning, deep learning, large language models, and ensemble algorithms consistently outperformed many conventional statistical techniques when evaluated using historical claims datasets. Retrieval-Augmented Generation (RAG) systems based on GPT-4o, Random Forest algorithms, and Genetic Support Vector Machines demonstrated high detection accuracy under controlled experimental conditions. Nevertheless, these findings should not be interpreted as evidence of equivalent performance in routine healthcare practice because most studies relied on retrospective datasets, institution-specific databases, and controlled analytical environments, limiting external validity (8, 25, 26, 30).

Several studies further demonstrated the ability of machine learning to identify complex fraud patterns that are difficult to detect using traditional auditing approaches. Multi-view bi-clustering successfully detected abnormal reimbursement behaviours, LSTM-based anomaly detection achieved stable predictive performance, and Patient Cluster Divergence and cost-sensitive learning models improved the identification of suspicious claims within large healthcare datasets. Although these findings indicate that machine learning substantially enhances fraud detection capability, most evaluations remain limited to retrospective or simulated datasets. Future research should therefore prioritise prospective validation studies, multicentre evaluations, and real-world implementation research to determine whether these promising results can be consistently replicated in operational health insurance systems (5, 12, 17, 19, 21).

##### 6.2.2.2 Blockchain and distributed architecture methodologies

Blockchain-based architectures have attracted considerable attention because of their potential to improve transparency, data integrity, and secure claims processing through decentralized ledger technologies and smart contracts. Several studies reported improvements in fraud detection accuracy and claim verification efficiency, particularly when blockchain was integrated with artificial intelligence or large language models. However, the available evidence is derived primarily from proof-of-concept implementations, prototype systems, or controlled experimental evaluations rather than large-scale operational deployments. Practical implementation remains constrained by interoperability with existing health information systems, infrastructure costs, computational requirements, regulatory compliance, organizational readiness, and governance challenges. Consequently, although blockchain represents a promising technological approach, stronger empirical evidence from routine healthcare settings is required before its effectiveness can be established with confidence. More recent experimental studies integrating blockchain with large language models have reported detection accuracies of up to 99% under controlled conditions, highlighting the potential of these technologies for real-time fraud monitoring. Nevertheless, these findings require further validation through prospective studies and large-scale operational implementation before they can be generalized to routine health insurance systems (19, 29, 30).

##### 6.2.2.3 Statistical and non-parametric methodologies

Statistical methods continue to play an important role in detecting irregular billing patterns. Quantile regression has proved useful for identifying aggressive billing practices that standard linear models often miss. Bayesian probability models offer a more reliable assessment of suspicious claims by assigning probability distributions rather than single-point estimates. Graph-based approaches have achieved high precision in outpatient claim analysis, while representation learning models have improved accuracy by filtering out unnecessary noise from clinical coding data (10, 13, 15, 16).

##### 6.2.2.4 Ethical, privacy, and implementation considerations

Despite the growing interest in AI- and blockchain-based fraud detection, several implementation challenges remain insufficiently addressed within the current literature. The use of large-scale healthcare datasets raises important concerns regarding patient privacy, data security, informed consent, and compliance with data protection regulations. AI models may also introduce algorithmic bias, reduce transparency in decision-making, and generate false-positive fraud alerts that require additional human verification. Similarly, blockchain-based systems face practical challenges related to scalability, interoperability with legacy healthcare information systems, implementation costs, governance, and regulatory acceptance. These issues suggest that technological innovation alone is insufficient for effective fraud prevention. Successful implementation will require robust governance frameworks, ethical oversight, appropriate regulatory safeguards, and continuous human supervision alongside technological solutions.

#### 6.2.3 Comparative synthesis of FWA control approaches

Strong governance and institutional interventions are essential for reducing fraud, waste, and abuse in health insurance. Effective monitoring, proper resource allocation, and financial oversight improve accountability and strengthen the financial stability of insurance systems. Some studies show that higher staffing levels and larger budgets for Medicaid Fraud Control Units (MFCUs) improve fraud detection and prosecution, as measured by increases in annual fraud exclusions. Effective risk governance is also strongly linked (*r* = 0.975) with maintaining a healthy Medicare fund balance. In the private sector, health insurance organizations using Payment Integrity Activities (PIA), such as targeted claim reviews, reported average savings of $4.8 million per organization. These findings show that strong institutional oversight can reduce financial losses and improve system efficiency (14, 22, 34).

#### 6.2.4 Policy, audit, and legislative methodologies

Policy-driven and audit-based approaches remain central to fraud control. Automated policy extraction systems help convert benefit rules into computable formats, making claim violations easier to identify. The Policy-Driven Health Insurance Fraud (PDHIF) methodology provides a structured framework that strengthens machine-learning-based detection. Legislative controls, such as Medical Loss Ratio reporting, also support oversight, although some evidence suggests manipulation in insurer reporting. In China, unannounced inspections and intelligent monitoring systems are increasingly used to strengthen supervision of medical insurance claims (5, 8, 12, 23, 27).

Legal frameworks and whistleblower protections play a central role in controlling fraud, waste, and abuse in health insurance by strengthening accountability and improving fraud detection. The False Claims Act (FCA), which prohibits the submission of false claims for government payments, is considered one of the oldest anti-fraud laws in the United States. Its effectiveness was further strengthened by the Affordable Care Act, which increased provider liability for failing to return overpayments and imposed strict financial penalties, including treble damages. Major settlements, such as the $1.7 billion paid by the Hospital Corporation of America, reflect its strong enforcement impact. Whistleblower protections under the FCA, particularly through qui tam provisions, have significantly improved fraud detection by allowing private individuals to report fraud on behalf of the government. In 2015, most of the Department of Justice’s healthcare fraud recoveries came through such cases, showing their financial importance. Other supporting laws, including the Anti-Kickback Statute, Stark Law, and Civil Monetary Penalty Statute, further prevent illegal referrals, unnecessary services, and false claims. In addition, the Exclusion Statute allows authorities to bar fraudulent providers from federal healthcare programs, reinforcing system integrity and discouraging repeated violations (11, 14, 44) (see Table 1).

**Table 1:** Studies that proposed FWA control interventions.

Study (country)	First author (year)	Technique	Type of fraud detected
Towards a Secure Technology-Driven Architecture for Smart Health Insurance Systems: An Empirical Study (Saudi Arabia)	Al-Quayed et al. (24)	Blockchain, 5G, cloud, and machine learning	Insurer, provider, and subscriber fraud
Multivariate Outlier Detection in Medicare Claims Payments Applying Probabilistic Programming Methods (United States)	Bauder et al. (10)	Outlier detection	Provider fraud
Ontology-Guided Policy Information Extraction for Healthcare Fraud Detection (Ireland)	Brisimi et al. (27)	Regulatory framework	Provider fraud
Medicare and the Affordable Care Act: Fraud Control Efforts and Results (United States)	Clemente et al. (11)	Policy framework	Subscriber and provider fraud
Accounting-Based Regulation: Evidence from Health Insurers and the Affordable Care Act (United States)	Eastman et al. (12)	Regulatory framework	Insurer fraud
Analysis of Health Care Billing via Quantile Variable Selection Models (United States)	Ekin et al. (13)	Statistical methods	Provider fraud
Impact of Enforcement on Healthcare Billing Fraud: Evidence from the United States (United States)	Flasher et al. (14)	Policy framework	Provider fraud
A Novel Multi-View Bi-Clustering Method for Identifying Abnormal Co-Occurrence Medical Visit Behaviours (China)	Guo et al. (19)	Data analytics	Subscriber fraud
Detecting Medical Prescriptions Suspected of Fraud Using an Unsupervised Data Mining Algorithm (Iran)	Haddad Soleymani et al. (32)	Data analytics	Medical prescription fraud
Identification of Fraudulent Healthcare Claims Using Fuzzy Bipartite Knowledge Graphs (United States)	Haque et al. (16)	Data analytics	Provider fraud
Identifying Health Insurance Claim Fraud Using a Mixture of Clinical Concepts (United States)	Haque et al. (15)	Data analytics	Provider fraud
Approaches for Identifying United States Medicare Fraud in Provider Claims Data (United States)	Herland et al. (17)	Machine learning and data mining	Provider fraud
Fraud Detection in Privacy Preserving Health Insurance System Using Blockchain Technology (Bangladesh)	Islam et al. (29)	Blockchain	Subscriber and provider fraud
Using Large Language Models for Enhanced Fraud Analysis and Detection in Blockchain-Based Health Insurance Claims (UAE, United States, Saudi Arabia)	Islayem et al. (25)	Blockchain and LLM	Provider fraud
Healthcare Insurance Frauds: Taxonomy and Blockchain-Based Detection Framework (Block-HI) (UAE, United States)	Ismail et al. (26)	Blockchain	Provider fraud
Multi-Stage Methodology to Detect Health Insurance Claim Fraud (United States)	Johnson et al. (18)	Analytical methods	Subscriber and provider fraud
Medblocksure: Blockchain-Based Insurance System (India)	Krishna et al. (30)	Blockchain	Subscriber and provider fraud
An Abnormal Surgical Record Recognition Model with Keyword Combination Patterns Based on Textrank for Medical Insurance Fraud Detection (China)	Li et al. (20)	Data analytics	Subscriber and provider fraud
AI-Driven Framework for Need-Based Insurance Plans Generation and Anomaly Detection Using Deep Learning Techniques (Pakistan)	Rehman et al. (5)	AI framework	Provider fraud
Decision Support System (DSS) for Fraud Detection in Health Insurance Claims Using Genetic Support Vector Machines (GSVMs) (Ghana)	Sowah et al. (28)	Data analytics and machine learning	Provider fraud
Patient Cluster Divergence-Based Healthcare Insurance Fraudster Detection (China)	Sun et al. (21)	Data analytics	Subscriber fraud
Research on the Risk of Fraudulent Reimbursement of Patient Consultation Fees (China)	Sun et al. (22)	Risk governance	Provider fraud
Outlier Detection in Healthcare Fraud: A Case Study in the Medicaid Dental Domain (The Netherlands)	Van Capelleveen et al. (42)	Data analytics	Provider fraud
Methodology for Detecting Suspicious Claims in Health Insurance Using Supervised Machine Learning (Peru)	Villegas-Ortega et al. (8)	Machine learning	Provider fraud
Research on Quantitative Evaluation of Medical Insurance Fraud Supervision Policy Based on “Antecedents-Process-Outcomes” Framework (China)	Zhang et al. (23)	Policy framework	Insurer, provider, and subscriber fraud
Big Data-Driven Abnormal Behaviour Detection in Healthcare Based on Association Rules (China)	Zhou et al. (45)	Data mining	Subscriber fraud

Overall, the reviewed evidence demonstrates that technological innovations alone are insufficient to achieve sustainable fraud control. Across countries, the most effective approaches combine advanced analytical tools with robust governance, regulatory oversight, institutional accountability, and legislative enforcement. This pattern suggests that technological solutions are most effective when implemented within comprehensive governance frameworks rather than as standalone interventions.

### 6.3 Impact on sustainability

While the reviewed studies consistently suggest that technological and governance interventions have the potential to improve the sustainability of health insurance systems, the strength of the available evidence varies considerably across study designs and healthcare contexts. Many reported benefits are based on modelling studies or controlled technical evaluations rather than long-term implementation studies. Consequently, the projected sustainability impacts should be interpreted as promising but not yet conclusive, highlighting the need for longitudinal evaluations in diverse healthcare systems.

#### 6.3.1 Financial sustainability and fund solvency

The findings of the present study show that FWA pose a serious threat to the long-term sustainability and financial stability of health insurance systems worldwide. These practices weaken fund solvency, disrupt financial balance, and reduce health systems’ ability to provide continuous services. Evidence from the United States shows that controlling healthcare FWA is essential for sustaining publicly funded programs, as fraud diverts resources and limits funding for other essential public services. Studies from China similarly identify FWA as a major risk to the safety and long-term solvency of medical insurance funds, with declining fund balance rates underscoring the need for stronger supervision and risk-prevention measures. The findings of the selected studies further indicate that effective risk governance plays a key role in maintaining durable healthcare funds and supporting the sustainable development of medical insurance systems (14, 22, 23, 45).

In the United States, Medicaid spending accounts for more than one-fourth of annual state budgets, and fraudulent activities further reduce the funds available for other essential public services. This makes effective fraud control necessary for maintaining the sustainability of state medical insurance programs. Research also shows that FWA threatens the long-term solvency of medical insurance funds by disturbing financial balance and affecting payment control systems. Evidence from China reflects this concern, where medical insurance fund balance rates declined from 23% in 2012 to 10% in 2018, highlighting the need for stronger management and monitoring systems. Similarly, in Indonesia, fraud is recognized as a direct threat to the sustainability of the National Health Insurance program and its goal of achieving universal health coverage (14, 23, 33, 45).

#### 6.3.2 Impact on system stability and viability

FWA creates serious threats to the stability and long-term viability of health insurance systems. The study’s findings show that the substantial financial losses from healthcare insurance fraud directly threaten the global sustainability of healthcare systems. In public insurance programs such as Medicare, continuous fraudulent activities can weaken program effectiveness and may even threaten their future viability if not controlled. Studies on blockchain-based insurance systems also note that fraud-related financial losses can jeopardize the overall stability of the healthcare industry. In the private insurance sector, widespread corruption and fraud can severely damage organizational performance, reduce service quality, and, in extreme cases, lead to regulatory closure or financial collapse of insurance firms (11, 26, 30, 34).

FWA leads to the misuse of healthcare resources, which undermines the financial stability and capability of health insurance systems. Research on Indonesia’s National Health Insurance program identifies hospital fraud as a major threat to the system’s financial feasibility. Findings from the studies also show that the diversion of resources through fraudulent claims weakens financial sustainability, erodes patient trust, and undermines the quality of healthcare services. Corruption further weakens health systems by disrupting progress toward universal health coverage and creating a cycle in which limited resources increase the risk of corruption, making funds even less available (5, 8, 33, 43). Additionally, the study by Sowah et al. (28) suggested that effective healthcare delivery systems are prioritized to sustain growth in low-income nations.

Morris links healthcare cost inflation to fraud, which ultimately results in the squandering of healthcare funds and the undermining of patient care (46). In losses caused by FWA, “The sum of the lowest available estimates exceeds 20% of total healthcare expenditures” (31). Berwick categorized waste into 6 categories and assessed the economic burden of each by calculating the annual cost to publicly funded insurance programs and the healthcare system as a whole: failures of care delivery, failures of care coordination, overtreatment, administrative complexity, pricing failures, and fraud and abuse.

When the FWA economic burden is identified, the future impact of such practices on the health system’s funding and financing is quantified (11). In theory, identifying the FWA’s economic burden enables financial prediction of its impact on health system funds and budgets; however, such a forecast is not as clear-cut as projecting financial losses from occasional errors. This challenge arises in the context of health insurance, specifically because fraudsters and abusers of healthcare funds nowadays have greater access to technologies that allow them to diversify their skill sets to accommodate and surpass tighter security measures aimed at controlling the inappropriate release of funds. Rapidly developing technologies create widening gaps that can be exploited by individuals or entities engaged in organized crime. Vian (43) elaborates on the impacts of FWA in terms of hindering access to care, financial protection of healthcare funds, and user satisfaction, in addition to the indirect influence on other determinants of health.

The misuse of healthcare resources through FWA has serious effects on both health systems and society. When funds are diverted through fraud, the cohesion of the healthcare systems is weakened, patient trust declines, and the quality of care may suffer. Corruption further weakens health systems by limiting their ability to support better health outcomes, economic development, and progress toward universal health coverage. Research also highlights a cycle in which limited resources increase the risk of corruption, while corruption further reduces the availability of funds for genuine healthcare needs. On the other hand, advanced fraud detection technologies can help prevent misuse of resources, improve financial stability, and strengthen the sustainability of insurance systems (11, 22, 26, 30) (see Table 2).

**Table 2:** Studies that address the impact of FWA on health system sustainability.

Study (country)	First author (year)	Projected sustainability impact
Towards a Secure Technology-Driven Architecture for Smart Health Insurance Systems: An Empirical Study (Saudi Arabia)	Al-Quayed et al. (24)	Lower health insurance premiums
Eliminating Waste in US Healthcare (United States)	Berwick et al. (31)	Waste reduction
Multivariate Outlier Detection in Medicare Claims Payments Applying Probabilistic Programming Methods (United States)	Bauder et al. (10)	Cost recovery
Accounting-Based Regulation: Evidence from Health Insurers and the Affordable Care Act (United States)	Eastman et al. (12)	Increased system integrity
A Novel Multi-View Bi-Clustering Method for Identifying Abnormal Co-Occurrence Medical Visit Behaviours (China)	Guo et al. (19)	Increased fund efficiency and effectiveness
Detecting Medical Prescriptions Suspected of Fraud Using an Unsupervised Data Mining Algorithm (Iran)	Haddad Soleymani et al. (32)	Efficiency and accuracy in fraud detection
Fraud Detection in a Privacy-Preserving Health Insurance System Using Blockchain Technology (Bangladesh)	Islam et al. (29)	Increased system integrity
Using Large Language Models for Enhanced Fraud Analysis and Detection in Blockchain-Based Health Insurance Claims (UAE, United States, Saudi Arabia)	Islayem et al. (25)	Reduction in financial loss
Potential of Hospital Fraud in the Indonesian National Health Insurance Era (A Descriptive Phenomenological Research) (Indonesia)	Khoiri et al. (33)	Increased fund efficiency
AI-Driven Framework for Need-Based Insurance Plans Generation and Anomaly Detection Using Deep Learning Techniques (Pakistan)	Rehman et al. (5)	Proper resource allocation
Preventing Fraud and Providing Services: The Private Healthcare Insurance Sector (United Kingdom)	Stiernstedt et al. (34)	Economic sustainability
Research on the Risk of Fraudulent Reimbursement of Patient Consultation Fees (China)	Sun et al. (22)	Economic sustainability
Anti-Corruption, Transparency, and Accountability in Health: Concepts, Frameworks, and Approaches (United States)	Vian (43)	Economic sustainability

#### 6.3.3 Demographic and social pressures

The impact of FWA becomes more serious as healthcare systems face growing demographic and social pressures. An aging global population increases the demand for healthcare services, placing greater strain on available resources and making fraud more harmful to fund security. The loss of healthcare funds through fraudulent claims also raises concerns of fairness, as resources meant for essential care are diverted away from genuine patients. This not only affects financial stability but also creates broader social challenges by reducing public confidence in healthcare systems (20, 22).

### 6.4 Publication trends

**Figure 2:**
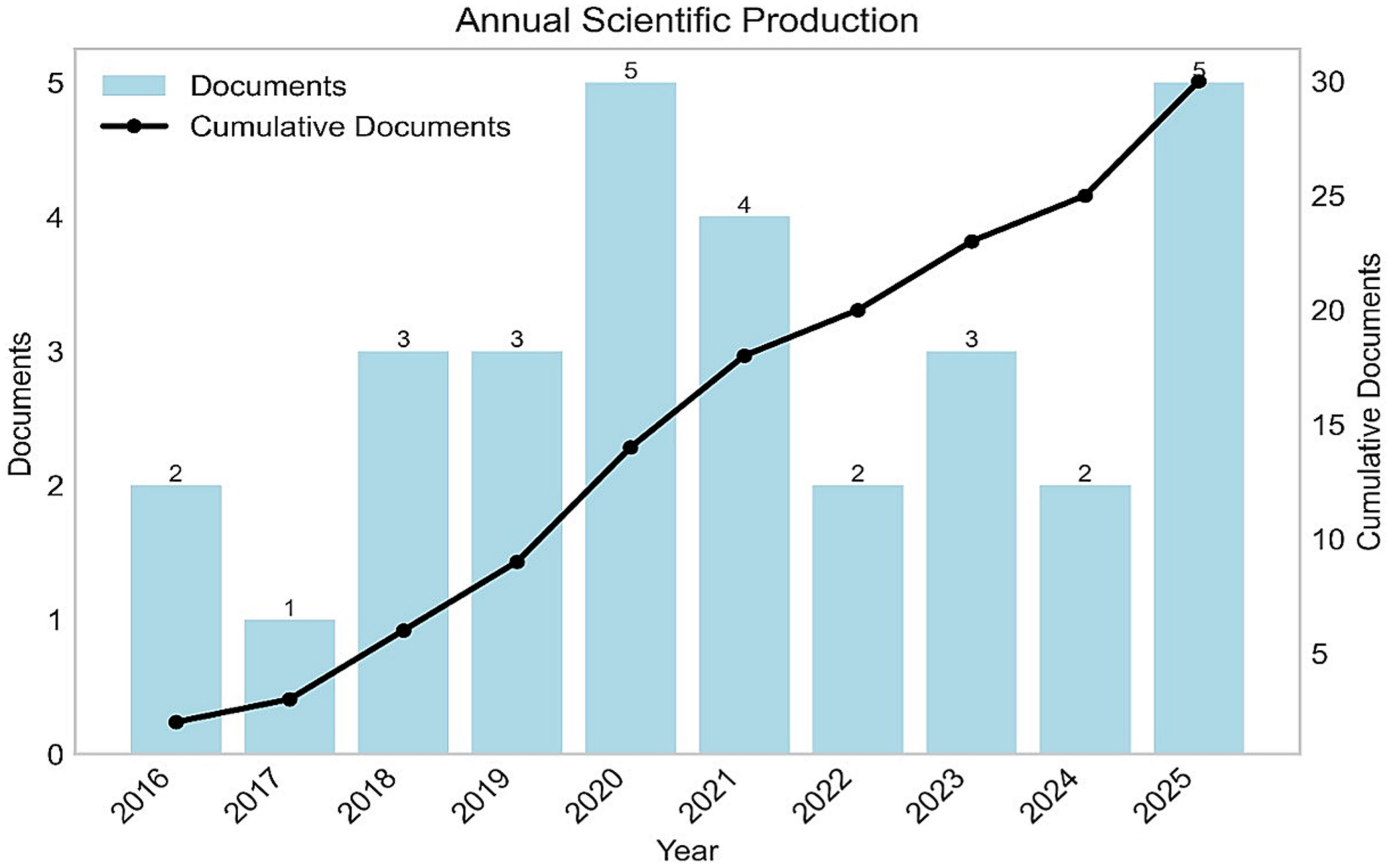
Year-wise publication trends.

The bibliometric analysis indicates a steady increase in research on the economic impact of fraud, waste, and abuse (FWA) in health insurance between 2016 and 2025, reflecting growing international recognition of the financial and policy implications of FWA. As shown in Figure 2, publication output increased gradually over the study period, with the highest productivity observed in 2020 and 2025, each contributing five publications. Overall, studies published during 2021–2025 accounted for 53% of the included literature, compared with 47% published during 2016–2020, demonstrating sustained growth in scholarly attention.

The increasing publication trend coincides with the rapid adoption of digital health technologies, growing concerns regarding healthcare financing, and heightened interest in strengthening the sustainability of health insurance systems. More recent studies have increasingly focused on artificial intelligence, machine learning, blockchain, and advanced data analytics as complementary approaches to traditional governance and regulatory interventions for detecting and preventing FWA. This evolution reflects a shift from describing the economic burden of FWA toward developing integrated technological and policy-based strategies for improving financial accountability and healthcare system resilience.

Despite this growth, the geographical distribution of the literature remains uneven. Most studies originated from high-income countries, particularly the United States and China, while evidence from low- and middle-income countries remains comparatively limited. This imbalance highlights the need for additional context-specific research to evaluate the feasibility, effectiveness, and sustainability of FWA control interventions in resource-constrained healthcare systems. Consequently, the observed publication trends not only demonstrate increasing academic interest but also reveal important gaps that should guide future research priorities.

**Figure 3:**
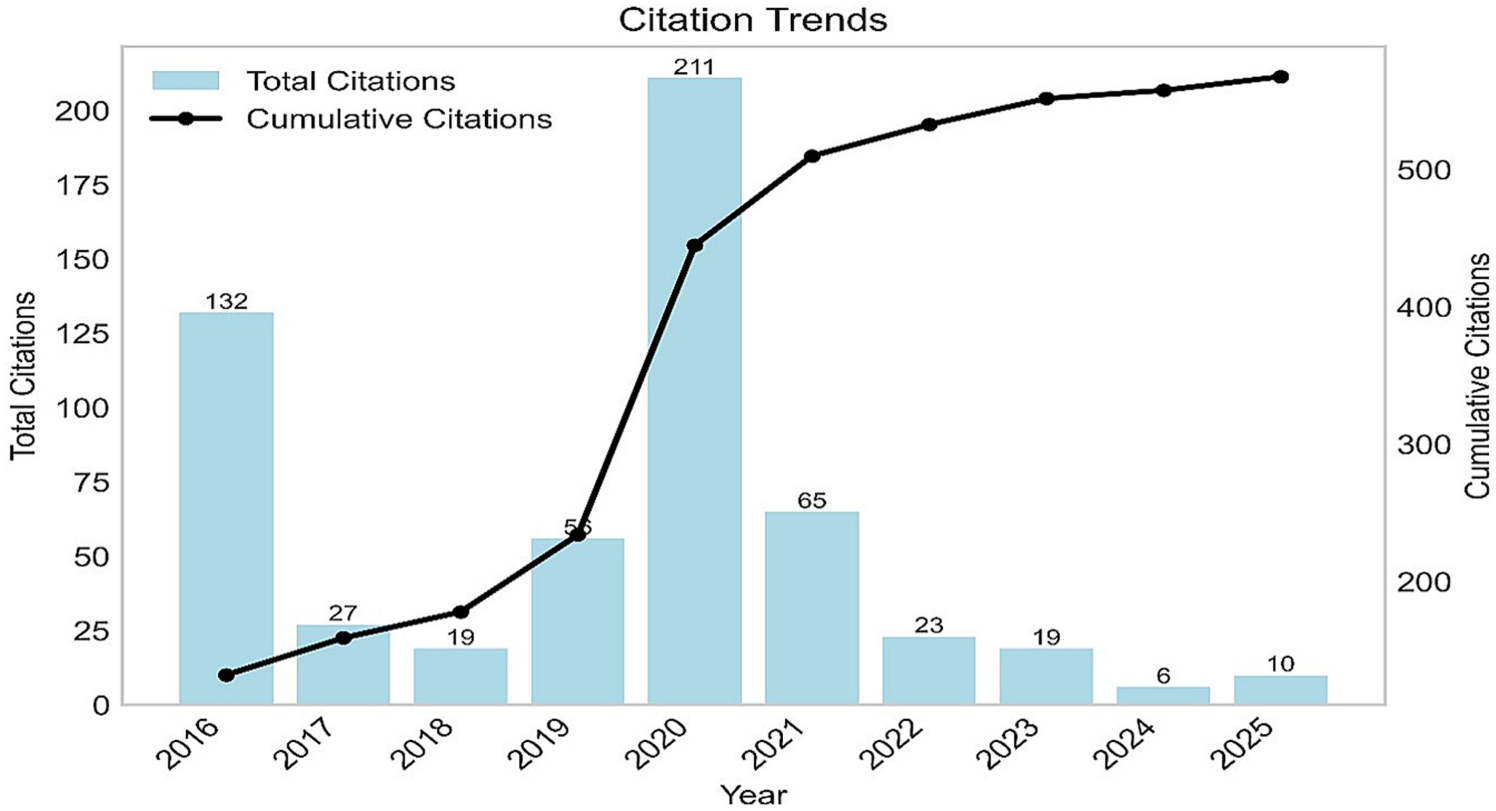
Year-wise citation trends.

Figure 3 demonstrates year-wise citations for scholarly publications between 2016 and 2025. The year 2020 led with 211 citations, followed by 2016 with 132 and 2019 with 56. Furthermore, the data analysis indicated that 2021 and 2017 obtained 65 and 27 citations, respectively. These 5 years have the highest number of citations, likely because the citation window is mature and the publications from these years may represent the core of the field.

In contrast, 2024 is the least-cited year, with 6 citations, followed by 2023 and 2025, which received 10 and 19 citations, respectively. These recent publications have few citations so far, a typical feature of recent work that takes time to be recognized.

The cumulative citation curve shows a strong cumulative impact, with a steady increase from 132 in 2016 to 568 in 2025, suggesting continued scholarly interest in the body of work. Significantly, the cumulative number for the first 5 years (2016–2020) accounts for 78% (445 of 568) of the total for the last 5 years, highlighting the value of early work, while the latter years have added incrementally, with a smaller increase in recent publications.

The country-wise production of studies on the economic burden of health insurance fraud, waste, and abuse from 2016 to 2025 is shown in Table 3. The United States ranks first with 16 publications, followed by China and Saudi Arabia with 6 and 3 publications, respectively. This is followed by Pakistan and the United Arab Emirates with 2 published studies each. On the other hand, in terms of the fewest publications, Bangladesh, Germany, Ghana, India, and Iran each produced 1 publication.

**Table 3:** Country-wise production.

Rank	Country	Publications
1	United States	16
2	China	6
3	Saudi Arabia	3
4	Pakistan	2
4	United Arab Emirates	2
5	Bangladesh	1
5	Germany	1
5	Ghana	1
5	India	1
5	Iran	1

### 6.5 Limitations

This review should be interpreted in light of several limitations. First, although a systematic literature search was undertaken across seven major bibliographic databases, the possibility of publication bias cannot be excluded because unpublished studies, government reports, dissertations, conference proceedings, and other forms of grey literature were not systematically included. Consequently, the findings primarily reflect evidence available in the published scientific literature.

Second, the review was restricted to English-language publications. Although this criterion ensured consistency in the review process, it may have resulted in the exclusion of relevant studies published in other languages, particularly those reporting experiences from non-English-speaking countries. This language restriction may have reduced the geographical diversity of the available evidence.

Third, the included studies exhibited substantial methodological and contextual heterogeneity. Differences in study design, healthcare systems, definitions of fraud, waste, and abuse (FWA), intervention types, outcome measures, and analytical methods limited direct comparison across studies and precluded quantitative meta-analysis. Consequently, the evidence was synthesized narratively, and the conclusions should be interpreted in the context of this heterogeneity.

Fourth, a formal methodological quality appraisal or quantitative risk-of-bias assessment of the included studies was not undertaken. Given the diversity of evidence, including empirical studies, technological evaluations, policy analyses, and review articles, the review focused on critically synthesizing the available literature rather than quantitatively evaluating study quality. Accordingly, variations in methodological rigor across individual studies should be considered when interpreting the findings (47).

Fifth, the available evidence was disproportionately derived from studies conducted in high-income countries, particularly the United States and China. Comparatively limited evidence was available from low- and middle-income countries, where differences in healthcare financing systems, regulatory capacity, digital infrastructure, and institutional resources may influence the feasibility and effectiveness of fraud detection interventions. Therefore, the generalizability of some findings to resource-constrained healthcare settings should be interpreted cautiously.

Finally, fraud detection technologies based on artificial intelligence, machine learning, blockchain, and advanced analytics continue to evolve rapidly. As the literature search included studies published up to 24 November 2025, subsequent technological developments, regulatory changes, and implementation experiences may not be reflected in this review. Future updates incorporating newly published evidence and formal methodological quality assessment would further strengthen the evidence base and provide a more comprehensive understanding of emerging approaches to combating fraud, waste, and abuse in health insurance systems.

## 7 Conclusion

Fraud, waste, and abuse (FWA) remain significant challenges for health insurance systems worldwide, resulting in substantial financial losses, inefficient allocation of healthcare resources, reduced fund solvency, and threats to the long-term sustainability of healthcare financing. This review demonstrates that effective FWA control requires a multidisciplinary approach integrating legislative enforcement, regulatory oversight, institutional governance, and advanced analytical technologies. While stronger legal frameworks, whistleblower protections, and institutional monitoring improve accountability and deter fraudulent activities, emerging technologies such as artificial intelligence, machine learning, blockchain, and automated auditing offer promising opportunities to strengthen fraud detection and claims verification.

The review further highlights that the impact of FWA extends beyond direct financial losses by contributing to resource misallocation, declining public trust, increased healthcare costs, and challenges to achieving universal health coverage. Although AI, machine learning, and blockchain technologies demonstrate substantial potential for improving fraud detection and financial accountability, the current evidence is derived predominantly from experimental studies and pilot implementations. Their successful translation into routine healthcare practice will depend on addressing challenges related to interoperability, scalability, ethical governance, regulatory compliance, privacy protection, implementation costs, and organizational readiness. Future research should therefore prioritize prospective implementation studies and comparative evaluations across diverse healthcare systems, particularly in low- and middle-income countries.

Overall, the evidence indicates that FWA should be viewed not only as a financial problem but also as a governance and sustainability challenge that directly influences health system performance. Policymakers should adopt integrated strategies that combine effective regulatory oversight, institutional accountability, technological innovation, and continuous monitoring to safeguard healthcare resources and improve system resilience. Continued international collaboration, investment in evidence-based fraud prevention strategies, and context-specific implementation research will be essential for strengthening the financial sustainability of health insurance systems and supporting progress toward universal health coverage.

## References

[1] Coyne JS Hilsenrath P. The world health report 2000: can health care systems be compared using a single measure of performance? Am J Public Health. (2002) 92:30–3. doi: 10.2105/ajph.92.1.30, 11772753 PMC1447380

[2] Kumaraswamy N Ekin T Park C Markey MK Barner JC Rascati K. Using a Bayesian belief network to detect healthcare fraud. Expert Syst Appl. (2023) 238:122241. doi: 10.1016/j.eswa.2023.122241

[3] Gee J. Button M. The financial cost of healthcare fraud 2014: what data from around the world shows. Report. (2014) Available online at: https://pure.port.ac.uk/ws/portalfiles/portal/17778625/The_Financial_Cost_of_Healthcare_Fraud_Report_2014_11.3.14a.pdf

[4] Simborg DW. Healthcare fraud: whose problem is it anyway? J Am Med Inform Assoc. (2008) 15:278–80. doi: 10.1197/jamia.m2672, 18308977 PMC2410006

[5] Rehman R Mazhar A Faheem A Matloob I Khan SA Shaukat S . Waste in the US health care system. JAMA. (2019) 322:1501. doi: 10.1001/jama.2019.13978, 31589283

[6] Glynn EH. Corruption in the health sector: A problem in need of a systems-thinking approach. Front Public Health. (2022) 10:910073. doi: 10.3389/fpubh.2022.910073, 36091569 PMC9449116

[7] Kruk ME Gage AD Arsenault C Jordan K Leslie HH Roder-DeWan S . High-quality health Systems in the Sustainable Development Goals era: time for a revolution. Lancet Glob Health. (2018) 6:e1196–252. doi: 10.1016/S2214-109X(18)30386-3, 30196093 PMC7734391

[8] Villegas-Ortega J Avilés LNQ Arancibia JN Montenegro WC Delgadillo R Quiroz Aviles LN . Methodology for detecting suspicious claims in health insurance using supervised machine learning. Future Internet. (2025) 17:584. doi: 10.3390/fi17120584

[9] Lee J Cho S. Abuse detection in healthcare insurance with disease-treatment network embedding. J Biomed Inform. (2021) 123:103936. doi: 10.1016/j.jbi.2021.103936, 34670175

[10] Bauder RA Khoshgoftaar TM. Multivariate outlier detection in Medicare claims payments applying probabilistic programming methods. Health Serv Outcomes Res Methodol. (2017) 17:256–89. doi: 10.1007/s10742-017-0172-1, 30311153

[11] Clemente S McGrady R Repass R Paul DP III Coustasse A. Medicare and the affordable care act: fraud control efforts and results. Int J Healthc Manag. (2018) 11:356–62. doi: 10.1080/20479700.2017.1401277

[12] Eastman EM Eckles DL Van Buskirk A. Accounting-based regulation: evidence from health insurers and the affordable care act. Account Rev. (2020) 96:231–59. doi: 10.2308/TAR-2019-0173

[13] Ekin T Damien P. Analysis of health care billing via quantile variable selection models. Healthcare. (2021) 9:1274. doi: 10.3390/healthcare9101274, 34682954 PMC8535243

[14] Flasher R Lamboy-Ruiz MA. Impact of enforcement on healthcare billing fraud: evidence from the USA. J Bus Ethics. (2019) 157:217–29. doi: 10.1007/s10551-017-3650-z

[15] Haque ME Tozal ME. Identifying health insurance claim fraud using a mixture of clinical concepts. IEEE Trans Serv Comput. (2022) 15:2356–67. doi: 10.1109/TSC.2021.3051165

[16] Haque ME Tozal ME. Identification of fraudulent healthcare claims using fuzzy bipartite knowledge graphs. IEEE Trans Serv Comput. (2023) 16:3931–45. doi: 10.1109/TSC.2023.3296782

[17] Herland M Bauder RA Khoshgoftaar TM. Approaches for identifying U.S. Medicare fraud in provider claims data. Health Care Manag Sci. (2020) 23:2–19. doi: 10.1007/s10729-018-9460-8, 30368641

[18] Johnson ME Nagarur N. Multi-stage methodology to detect health insurance claim fraud. Health Care Manag Sci. (2016) 19:249–60. doi: 10.1007/s10729-015-9317-3, 25600704

[19] Guo Y Zheng Z Kong L Guo W Yan Z Cui L . A novel multi-view bi-clustering method for identifying abnormal co-occurrence medical visit behaviors. Methods. (2022) 207:65–73. doi: 10.1016/j.ymeth.2022.09.004, 36122881

[20] Li W Ye P Yu K Min X Xie W. An abnormal surgical record recognition model with keyword combination patterns based on TextRank for medical insurance fraud detection. Multim Tools Appl. (2023) 82:30949–63. doi: 10.1007/s11042-023-14529-4

[21] Sun C Li Q Li H Shi Y Zhang S Guo W. Patient-cluster divergence-based healthcare insurance fraudster detection. IEEE Access. (2018) 7:14162–70. doi: 10.1109/access.2018.2886680

[22] Sun J Wang Y Zhang Y Li L Li H Liu T . Research on the risk governance of fraudulent reimbursement of patient consultation fees. Front Public Health. (2024) 12:1339177. doi: 10.3389/fpubh.2024.1339177, 38410668 PMC10895054

[23] Zhang Z Ding S Yang Z Hu H. Research on quantitative evaluation of medical insurance fraud supervision policy based on the ‘antecedents-process-outcomes’ framework. PLoS One. (2025) 20:e0313618. doi: 10.1371/journal.pone.0313618 39761318 PMC11703012

[24] Al-Quayed F Humayun M Tahir S. Towards a secure technology-driven architecture for smart health insurance systems: an empirical study. Healthcare. (2023) 11:2257. doi: 10.3390/healthcare11162257, 37628455 PMC10454849

[25] Islayem R Gebreab S AlKhader W Musamih A Salah K Jayaraman R . Using large language models for enhanced fraud analysis and detection in Blockchain-based health insurance claims. Sci Rep. (2025) 15:29763. doi: 10.1038/s41598-025-15676-4, 40804301 PMC12350640

[26] Ismail L Zeadally S. Healthcare insurance frauds: taxonomy and blockchain-based detection framework (block-HI). IT Prof. (2021) 23:36–43. doi: 10.1109/mitp.2021.3071534

[27] Brisimi TS Lopez V Rho V Sbodio ML Picco G Kristiansen M . Ontology-guided policy information extraction for healthcare fraud detection. Stud Health Technol Inform. (2020) 270:879–83. doi: 10.3233/SHTI200287 32570508

[28] Sowah RA Kuuboore M Ofoli A Kwofie S Asiedu L Koumadi KM . Decision support system (DSS) for fraud detection in health insurance claims using genetic support vector machines (GSVMS). J Eng. (2019) 20:1–19. doi: 10.1155/2019/1432597

[29] Islam MM Ahmed M Palit R Rahman MS Islam S. Fraud detection in privacy preserving health insurance system using blockchain technology. Eng Rep. (2025) 7:70315. doi: 10.1002/eng2.70315

[30] Krishna C Kumar D Kushwaha DS. MedBlockSure: blockchain-based insurance system. Cogn Comput Syst. (2024) 6:98–107. doi: 10.1049/ccs2.12112

[31] Berwick DM Hackbarth AD. Eliminating waste in US health care. JAMA. (2012) 307:1513–6. doi: 10.1001/jama.2012.362, 22419800

[32] Haddad Soleymani M Yaseri M Farzadfar F Mohammadpour A Sharifi F Kabir MJ. Detecting medical prescriptions suspected of fraud using an unsupervised data mining algorithm. DARU. (2018) 26:209–14. doi: 10.1007/s40199-018-0227-z, 30460618 PMC6279664

[33] Khoiri A Hidayat W Chalidyanto D Suhariadi F. Potential of hospital fraud in the Indonesian National Health Insurance era (a descriptive phenomenological research). Indian J Forensic Med Toxicol. (2020). doi: 10.37506/ijfmt.v14i3.10527

[34] Stiernstedt P Brooks G. Preventing fraud and providing services: the private healthcare insurance sector. Secur J. (2021) 34:621–34. doi: 10.1057/s41284-020-00252-4

[35] Gee J. Button M. Brooks G. The Financial Cost of Healthcare Fraud London MacIntyre Hudson/CCFS (2010) Available online at: https://storagehub.homnya.com/cmsimage/allegati/allegato6444539.pdf

[36] Leder-Luis J Malani A. The Economics of Healthcare Fraud. Cambridge: National Bureau of Economic Research (2025).

[37] Thaifur AYBR Maidin MA Sidin AI Razak A. How to detect healthcare fraud? A systematic review. Gac Sanit. (2021) 35:S441–9. doi: 10.1016/j.gaceta.2021.07.022 34929872

[38] Ai J Russomanno J Guigou S Allan R. A systematic review and qualitative assessment of fraud detection methodologies in health care. N Am Actuar J. (2021) 26:1–26. doi: 10.1080/10920277.2021.1895843

[39] Li J Huang K Jin J Shi J. A survey on statistical methods for health care fraud detection. Health Care Manag Sci. (2008) 11:275–87. doi: 10.1007/s10729-007-9045-4, 18826005

[40] Amponsah AA Adekoya AF Weyori BA. Improving the financial security of National Health Insurance using cloud-based blockchain technology application. Int J Inf Manag Data Insights. (2022) 2:100081. doi: 10.1016/j.jjimei.2022.100081

[41] Habib SS Perveen S Khuwaja HMA. The role of Micro health insurance in providing financial risk protection in developing countries: a systematic review. BMC Public Health. (2016) 16:281. doi: 10.1186/s12889-016-2937-9, 27004824 PMC4802630

[42] Van Capelleveen G Poel M Mueller RM Thornton D Van Hillegersberg J. Outlier detection in healthcare fraud: a case study in the Medicaid dental domain. Int J Account Inf Syst. (2016) 21:18–31. doi: 10.1016/j.accinf.2016.04.001

[43] Vian T. Anti-corruption, transparency, and accountability in health: concepts, frameworks, and approaches. Glob Health Action. (2020) 13:1694744. doi: 10.1080/16549716.2019.1694744, 32194010 PMC7170369

[44] McGee J Sandridge L Treadway C Vance K Coustasse A. Strategies for fighting Medicare fraud. Health Care Manag. (2018) 37:147–54. doi: 10.1097/hcm.0000000000000204, 29697574

[45] Zhou S He J Yang H Chen D Zhang R. Big data-driven abnormal behavior detection in healthcare based on association rules. IEEE Access. (2020) 8:129002–11. doi: 10.1109/access.2020.3009006

[46] Morris L. Combating fraud in health care: an essential component of any cost containment strategy. Health Aff. (2009) 28:1351–6. doi: 10.1377/hlthaff.28.5.1351, 19738251

[47] Greenhalgh T Wong G Westhorp G Pawson R. Protocol-realist and Meta-narrative evidence synthesis: evolving standards (RAMESES). BMC Med Res Methodol. (2011) 11:115. doi: 10.1186/1471-2288-11-115, 21843376 PMC3173389

[48] Page MJ McKenzie JE Bossuyt PM Boutron I Hoffmann TC Mulrow CD . The PRISMA 2020 statement: an updated guideline for reporting systematic reviews. BMJ. (2021) 372:n71. Available online at: https://www.prisma-statement.org., 33782057 10.1136/bmj.n71 PMC8005924

