# Supplementary material for: Economic impact of health insurance fraud, waste, and abuse: a literature review of direct and indirect costs and system sustainability
Source: Front Public Health. 2026 Jul 30;14:1883020. doi: 10.3389/fpubh.2026.1883020 (PMC13467992; doi:10.3389/fpubh.2026.1883020)
Supplement: Supplementary file 1 [file Supplementary_file_1.docx]

**Search strategy**

**1) Scopus (TITLE-ABS-KEY)**

TITLE-ABS-KEY(

  ("health economic*" OR "healthcare economic*" OR "health care economic*"

   OR "healthcare finance*" OR "health care finance*" OR "health financing"

   OR "healthcare financing" OR "health care financing"

   OR "healthcare funding" OR "health care funding"

   OR "healthcare sustainab*" OR "health system sustainab*" OR "financial sustainab*"

   OR solvency OR "budget impact" OR expenditure* OR spending OR "economic burden")

)

AND

TITLE-ABS-KEY(

  ("health insurance fraud" OR "medical insurance fraud" OR "insurance fraud"

   OR "insurance abuse" OR "insurance waste"

   OR "fraud waste abuse" OR "fraud, waste and abuse" OR FWA

   OR "improper payment*" OR overpayment* OR "false claim*" OR "fraudulent claim*"

   OR upcod* OR overbilling OR miscoding OR "phantom billing" OR "ghost billing"

   OR kickback* OR "duplicate claim*")

)

AND

TITLE-ABS-KEY(

  ("direct cost*" OR "indirect cost*" OR "financial loss*" OR "fraud loss*"

   OR "economic burden" OR "budget impact" OR sustainab* OR solvency)

)

**2) Web of Science (TS=)**

TS=(

  ("health economic*" OR "healthcare finance*" OR "health financing" OR "healthcare funding"

   OR "healthcare sustainab*" OR "health system sustainab*" OR "financial sustainab*"

   OR solvency OR "budget impact" OR expenditure* OR spending OR "economic burden")

  AND

  ("health insurance fraud" OR "insurance fraud" OR "insurance abuse" OR "insurance waste"

   OR "fraud, waste and abuse" OR "fraud waste abuse" OR FWA

   OR "improper payment*" OR overpayment* OR "false claim*" OR upcod*

   OR overbilling OR miscoding OR "phantom billing" OR kickback* OR "duplicate claim*")

  AND

  ("direct cost*" OR "indirect cost*" OR "financial loss*" OR "fraud loss*" OR "economic burden"

   OR "budget impact" OR sustainab* OR solvency)

)

**3) CINAHL (EBSCO) (TX)**

TX(

  ("health economic*" OR "healthcare finance*" OR "health financing" OR "healthcare funding"

   OR "healthcare sustainab*" OR "health system sustainab*" OR "financial sustainab*"

   OR solvency OR "budget impact" OR expenditure* OR spending OR "economic burden")

  AND

  ("health insurance fraud" OR "insurance fraud" OR "insurance abuse" OR "insurance waste"

   OR "fraud, waste and abuse" OR FWA OR "improper payment*" OR overpayment*

   OR "false claim*" OR upcod* OR overbilling OR miscoding OR "phantom billing"

   OR kickback* OR "duplicate claim*")

  AND

  ("direct cost*" OR "indirect cost*" OR "financial loss*" OR "fraud loss*" OR "economic burden"

   OR "budget impact" OR sustainab* OR solvency)

)

**4) PubMed and MEDLINE (MeSH + keywords)**

(

  ("Health Expenditures"[MeSH] OR "Costs and Cost Analysis"[MeSH] OR "Health Care Costs"[MeSH]

   OR "Financing, Organized"[MeSH] OR "Economics, Medical"[MeSH] OR "Insurance, Health"[MeSH]

   OR health econom*[tiab] OR healthcare finance*[tiab] OR health financ*[tiab]

   OR healthcare funding[tiab] OR "financial sustainab*"[tiab] OR "health system sustainab*"[tiab]

   OR solvency[tiab] OR "budget impact"[tiab] OR expenditure*[tiab] OR spending[tiab] OR "economic burden"[tiab]

  )

  AND

  ("Fraud"[MeSH] OR "Insurance Claim Review"[MeSH]

   OR "health insurance fraud"[tiab] OR "medical insurance fraud"[tiab] OR "insurance fraud"[tiab]

   OR "insurance abuse"[tiab] OR "insurance waste"[tiab]

   OR "fraud, waste and abuse"[tiab] OR FWA[tiab]

   OR "improper payment*"[tiab] OR overpayment*[tiab] OR "false claim*"[tiab]

   OR upcod*[tiab] OR overbilling[tiab] OR miscoding[tiab]

   OR "phantom billing"[tiab] OR "duplicate claim*"[tiab] OR kickback*[tiab]

  )

  AND

  ("direct cost*"[tiab] OR "indirect cost*"[tiab] OR "financial loss*"[tiab] OR "fraud loss*"[tiab]

   OR "economic burden"[tiab] OR "budget impact"[tiab] OR sustainab*[tiab] OR solvency[tiab]

  )

)

**5) Embase, OpenAlex, and Dimensions (Emtree + ti,ab,kw)**

(

  'health economics'/exp OR 'health care cost'/exp OR 'cost analysis'/exp

  OR 'health care financing'/exp OR 'health insurance'/exp OR 'budget'/exp

  OR health econom*:ti,ab,kw OR healthcare finance*:ti,ab,kw OR health financ*:ti,ab,kw

  OR "healthcare funding":ti,ab,kw OR "financial sustainab*":ti,ab,kw OR "health system sustainab*":ti,ab,kw

  OR solvency:ti,ab,kw OR "budget impact":ti,ab,kw OR expenditure*:ti,ab,kw OR spending:ti,ab,kw OR "economic burden":ti,ab,kw

)

AND

(

  'fraud'/exp OR 'insurance fraud'/exp

  OR "health insurance fraud":ti,ab,kw OR "medical insurance fraud":ti,ab,kw OR "insurance fraud":ti,ab,kw

  OR "insurance abuse":ti,ab,kw OR "insurance waste":ti,ab,kw

  OR "fraud, waste and abuse":ti,ab,kw OR FWA:ti,ab,kw

  OR "improper payment*":ti,ab,kw OR overpayment*:ti,ab,kw OR "false claim*":ti,ab,kw

  OR upcod*:ti,ab,kw OR overbilling:ti,ab,kw OR miscoding:ti,ab,kw OR "phantom billing":ti,ab,kw

  OR "duplicate claim*":ti,ab,kw OR kickback*:ti,ab,kw

)

AND

(

  "direct cost*":ti,ab,kw OR "indirect cost*":ti,ab,kw OR "financial loss*":ti,ab,kw OR "fraud loss*":ti,ab,kw

  OR "economic burden":ti,ab,kw OR "budget impact":ti,ab,kw OR sustainab*:ti,ab,kw OR solvency:ti,ab,kw

)

**6) Livivo (simple advanced query)**

Livivo supports Boolean but can be less strict. Use a compact version:

("health economics" OR "healthcare finance" OR "health financing" OR "healthcare funding" OR "financial sustainability" OR solvency OR "budget impact" OR expenditure OR spending)

AND

("health insurance fraud" OR "insurance fraud" OR "fraud, waste and abuse" OR FWA OR "improper payment" OR overpayment OR upcoding OR overbilling OR miscoding OR "phantom billing" OR "false claim" OR kickback OR "duplicate claim")

AND

("direct cost" OR "indirect cost" OR "financial loss" OR "fraud loss" OR "economic burden")
